# Hearing Loss and Cognition Among Older Adults in a Han Chinese Cohort

**DOI:** 10.3389/fnins.2019.00632

**Published:** 2019-06-25

**Authors:** Fuxin Ren, Jianfen Luo, Wen Ma, Qian Xin, Lei Xu, Zhaomin Fan, Yu Ai, Bin Zhao, Fei Gao, Haibo Wang

**Affiliations:** ^1^Shandong Medical Imaging Research Institute, Shandong University, Jinan, China; ^2^Department of Otolaryngology-Head and Neck Surgery, Shandong Provincial ENT Hospital, Shandong University, Jinan, China; ^3^Shandong Provincial Key Laboratory of Otology, Jinan, China; ^4^Department of Otolaryngology, Jinan Central Hospital, Shandong University, Jinan, China; ^5^The Second Hospital of Shandong University, Jinan, China

**Keywords:** presbycusis, hearing loss, cognition, older adults, speech reception thresholds

## Abstract

Presbycusis (PC) is associated with cognitive decline and incident dementia. Speech reception thresholds (SRT) are used to assess speech detection, which points toward a central component of PC. However, to the best of our knowledge, no previous study has reported the relationship between SRT and cognitive function in older adults in a Han Chinese cohort. Therefore, in this study, we investigate the association of hearing loss, indexed using pure tone average (PTA) and SRT, with cognitive function in a Han Chinese cohort using a standardized neurocognitive battery. Subjects (aged ≥60 years) with no history of psychiatric or neurological diseases were recruited. All subjects underwent a battery of neuropsychological and auditory tests. According to the PTA of the better ear, the subjects were further divided into PC and normal PTA (NP) groups. Regression analyses were performed to examine the relationship between cognitive function and hearing loss in the PC and NP groups and all subjects when controlling for age, sex, education level, diabetes, smoking, and hypertension. Cognitive function was significantly associated with PTA and SRT in all subjects. In all subjects, the correlations between non-verbal cognitive scores and SRT were stronger than those between non-verbal cognitive scores and PTA, whereas the correlations between verbal cognitive scores and PTA were stronger than those between verbal cognitive scores and SRT. Moreover, the correlations between PTA or SRT and cognitive function in the PC group were in principle stronger than those in the NP group. Our findings indicate that cognitive function is significantly associated with PTA and SRT in older adults in a Han Chinese cohort. Therefore, SRT could be an important auditory test for exploring cognitive decline in PC and could complement PTA.

## Introduction

Age-related hearing loss, also known as presbycusis (PC), is among the most common chronic diseases in older adults. The prevalence of PC increases with age, and it has been reported that 30–35% of adults between the ages of 65–75 years and 40–50% of adults over the age of 75 years suffer from PC (Nash et al., 2011). The World Health Organization’s World Health Statistics report indicated that the average life expectancy in China has increased significantly in recent years (2018). Age-related hearing loss is becoming a major sensory deficit among older adults in China (Luo et al., 2013). Recently, many audiological studies have suggested that PC is associated with cognitive impairment and incident dementia (Gates and Mills, 2005; Lin, 2011; Lin et al., 2011a,b,c; Jayakody et al., 2018). For instance, one cross-sectional study reported that hearing loss of older American adults is independently associated with lower scores on the symbol digit modalities test (SDMT) (Lin, 2011). In addition, reduced cognitive functioning was associated with age-related hearing loss in older European adults (Quaranta et al., 2014). However, to date, there has been a paucity of studies exploring the association between hearing loss and cognitive function in a Han Chinese cohort.

Presbycusis is usually characterized by progressive hearing loss at high frequencies, which are particularly important for speech recognition. Previous studies have shown that PC represents deteriorated function of both the auditory periphery and the central auditory system (Ouda et al., 2015). Hypofunction of the inner ear is the main reason for the peripheral component of PC (Schuknecht and Gacek, 1993). Moreover, poor speech discrimination and deteriorated temporal sound processing reflect a possible central component of PC (Mazelova et al., 2003). However, research into the association between hearing loss and cognitive function has focused solely on hearing as measured by pure tone thresholds, which assess the function of the auditory periphery. Speech reception thresholds (SRT) are used to assess speech detection, which could reflect a central component of PC. To our knowledge, to date there has been a paucity of studies exploring the relationship between SRT and cognitive function in older adults.

Therefore, in the present study, we investigated the association of hearing loss with cognitive function in a Han Chinese cohort using a standardized neurocognitive battery. Hearing loss was assessed using pure tone thresholds and SRT. We hypothesized that cognitive decline would be associated with both pure tone thresholds and SRT.

## Materials and Methods

### Subjects

The study was approved by the Shandong University institutional review board, and each participant provided informed consent. Subjects aged ≥60 years were recruited from the local community. No one had a history of psychiatric or neurological illness. All subjects were Han Chinese.

### Assessment of Auditory Function

First, optimal middle ear conditions were confirmed using tympanometry performed with a GSI Tympstar. Then, the air conduction thresholds were measured through pure tone audiometry at the frequencies from 125, 250, 500, 1000, 2000, 4000, and 8000 Hz using a clinical audiometer (GSI AudioStar Pro). The speech-frequency pure-tone average (PTA) for each side of ears was obtained by calculating the average of hearing thresholds at 500, 1000, 2000, and 4000 Hz in air conduction (1). Hearing loss was defined as PTA >25 decibels hearing level (dB HL) for the better ear (World Health Organization [WHO], 2018), and the subjects were further divided into PC and normal PTA (NP) groups. The exclusion criteria in the study were as follows: (1) asymmetric hearing loss, conductive hearing loss and tinnitus; (2) other causes of sensorineural hearing loss different from PC; (3) previous history of hearing aid use, noise exposure, otologic surgery, head trauma and ototoxic drug therapy.

Speech reception thresholds was performed with a clinical audiometer (GSI AudioStar Pro) and equipped with TDH-50P headphones in quiet conditions. First, according to the value of PTA, an initial sound intensity is groped to ensure that the subject is able to exactly recognize 5 spondee words under this intensity. If the subject cannot recognize, the software will increase the initial sound intensity. The software then automatically controls sound intensity: the sound intensity reduces by 5 dB for every five words played. The test is stopped when the subject is unable to exactly recognize five spondee words. Finally, the number of exact recognized words are counted in the whole descending process, then the software subtracts the number of exact recognized words from initial sound intensity and adds a correction factor (2.5 dB), which is the subject’s speech recognition threshold.

### Assessment of Cognitive Function

First, the subjects’ general cognitive function was tested using the Montreal Cognitive Assessment (MoCA) (Galea and Woodward, 2005; Nasreddine et al., 2005). Then, the subjects’ verbal learning and memory, attention, psychomotor speed and executive control were tested using the Auditory Verbal Learning Test (AVLT, Chinese version) (Zhao et al., 2012), the Stroop color word interference test (Savitz and Jansen, 2003), the SDMT (Van Schependom et al., 2014), and the trail-making test (TMT) (Sanchez-Cubillo et al., 2009), respectively. The TMT is made up of two parts. In TMT-A part, the subjects were required to draw lines sequentially connecting 25 encircled numbers, that were distributed on a piece of paper in ascending order (from 1 to 25), as soon as possible. Culturally, most Chinese older adults are less familiar with Arabic numerals than Westerners, therefore, a Chinese version of the TMT-B was applied in our study (Lu et al., 2006; Wei et al., 2018). In TMT-B part, the subjects were required to draw a line alternating between squares and circles while connecting the numbers on a page of the same size in ascending order (from 1 to 25) as soon as possible. Cognitive tests in our study were divided into two categories: verbal tests and non-verbal tests. Verbal tests include MoCA, AVLT and Stroop, which means that verbal communication is required in the test. Non-verbal tests include SDMT, TMT-A, and TMT-B, which means that verbal communication is not required in the test. Finally, the subjects’ anxiety and depression status were assessed using the hospital anxiety and depression scale (HADS) (Zigmond and Snaith, 1983). The overall testing time was about 60 min in a fixed order.

### Statistical Analysis

The two-tailed *t*-test was used to assess group differences in continuous variables, and the chi-square test was used to assess group differences in gender-specific, diabetes, smoking and hypertension. Partial correlation analyses were used to explore the correlations between cognitive function and PTA or SRT in the PC and NP groups and all subjects, controlling for age, sex, education level, diabetes, smoking and hypertension. Stepwise Multiple Linear Regression analyses were then performed in the PC group, NP group and all subjects. PTA/SRT, age, sex, education level, diabetes, smoking, and hypertension are independent variables, and cognition measures are dependent variables. *P*-values of less than 0.05 were accepted as significant.

## Results

### Demographic and Clinical Characteristics

The demographic and clinical characteristics of the study population are listed in Table 1. Eighty subjects aged 60–78 years (40 males/40 females, mean age: 65.78 ± 3.96 years) were screened for inclusion in this study. Thirty-five subjects were assigned to the NP group, and forty-five subjects were assigned to the PC group.

**Table 1:** Subjects’ demographic and clinical data.

Characteristics	All subjects	PC group	NP group	*P*-value PC vs. NP	Cohen’s d PC vs. NP
	(*n* = 80)	(*n* = 45)	(*n* = 35)		
Gender (male/female)	40/40	23/22	17/18	0.822	–
Age (years)	65.78 ± 3.96	66.29 ± 4.37	65.11 ± 3.30	0.190	0.30
Education (years)	11.50 ± 2.61	11.42 ± 2.35	11.60 ± 2.94	0.771	**–**0.07
Diabetes (yes/no)	7/73	4/41	3/32	0.960	–
Smoking (yes/no)	21/59	12/33	9/26	0.923	–
Hypertension(yes/no)	39/41	22/23	17/18	0.978	–
Anxiety	3.26 ± 2.40	3.38 ± 2.59	3.11 ± 2.15	0.629	0.11
Depression	3.38 ± 2.83	3.62 ± 3.43	3.06 ± 1.78	0.344	0.20
PTA (dB/HL)	25.97 ± 13.92	35.97 ± 10.18	13.11 ± 3.67	**<0.001**	**2.98**
SRT (dB/HL)	25.93 ± 13.99	35.43 ± 11.16	13.70 ± 4.49	**<0.001**	**2.48**
MoCA	25.68 ± 2.23	25.24 ± 2.79	26.23 ± 0.97	**0.032**	**–0.51**
AVLT	56.63 ± 8.63	55.58 ± 7.90	57.97 ± 9.44	0.232	**–**0.27
Stroop (s)	131.50 ± 16.20	135.13 ± 16.66	126.83 ± 14.52	**0.022**	**0.53**
SDMT	39.50 ± 15.12	34.71 ± 12.81	45.66 ± 15.80	**0.001**	**–0.76**
TMT-A (s)	48.86 ± 16.91	52.60 ± 16.89	44.06 ± 15.91	**0.024**	**0.53**
TMT-B (s)	140.48 ± 83.65	150.44 ± 85.40	127.66 ± 80.75	0.229	0.27


*The data are presented as means ± standard deviations. Values in bold are statistically significant. Cohen’s d is an effect size used to indicate the standardized difference between two means. PTA, pure tone average; SRT, speech reception threshold; PC, presbycusis; NP, normal PTA; MoCA, montreal cognitive assessment; AVLT, auditory verbal learning test; SDMT, symbol digit modalities test; TMT, trail-making test; levels of anxiety and depression were assessed according to the hospital anxiety and depression scale (HADS).*

**FIGURE 1:**
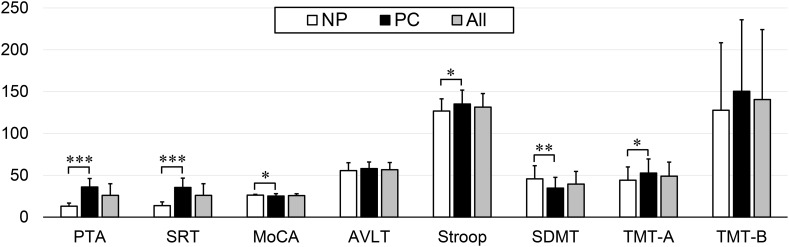
Differences in the neuropsychological and auditory tests between the Normal PTA (NP) group and Presbycusis (PC) group. Compared to the NP group, the patients with PC performed worse on the MoCA, Stroop, SDMT, and TMT-A tests (*p* < 0.05). The PTA and SRT were significantly higher in the patients with PC than in the NP group (*p* < 0.001). Statistical significance was set as ^∗^*p* < 0.05, ^∗∗^*p* < 0.01, and ^∗∗∗^*p* < 0.001.

**FIGURE 2:**
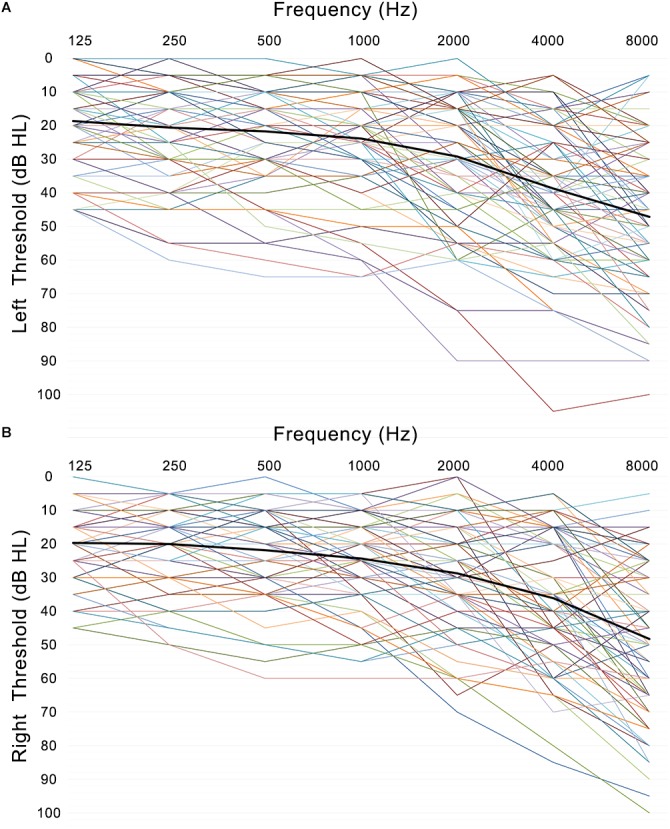
Left **(A)** and right **(B)** ear pure-tone thresholds (dB HL) are plotted for each subject’s left and right ear as a function of frequency (Hz) with the mean thresholds across participants and ears (black line).

Presbycusis and NP groups did not differ significantly in age, sex or education. Compared to the NP group, the patients with PC performed worse on the MoCA, Stroop, SDMT, and TMT-A tests (*p* < 0.05) (Table 1 and Figure 1). The correlations between cognitive outcomes in all subjects, NP and PC group were shown in Supplementary Table S1. The PTA and SRT were significantly higher in the patients with PC than in the NP group (PTA, *p* < 0.001; SRT, *p* < 0.001) (Table 1 and Figure 1). All subjects had a type A curve on tympanometry, which indicate normal middle ear function. The hearing thresholds of the left and right ears of all subjects are shown in Figure 2. Partial correlation analyses (controlling for age, sex, education level, diabetes, smoking and hypertension) revealed that SRT was positively correlated with PAT in all subjects (*r* = 0.945, *p* < 0.001), in the NP group (*r* = 0.472, *p* = 0.013) and in the PC group (*r* = 0.897, *p* < 0.001), as seen in Supplementary Figure S1.

### Correlations Between Hearing Loss and Cognitive Function (Table 2)

**FIGURE 3:**
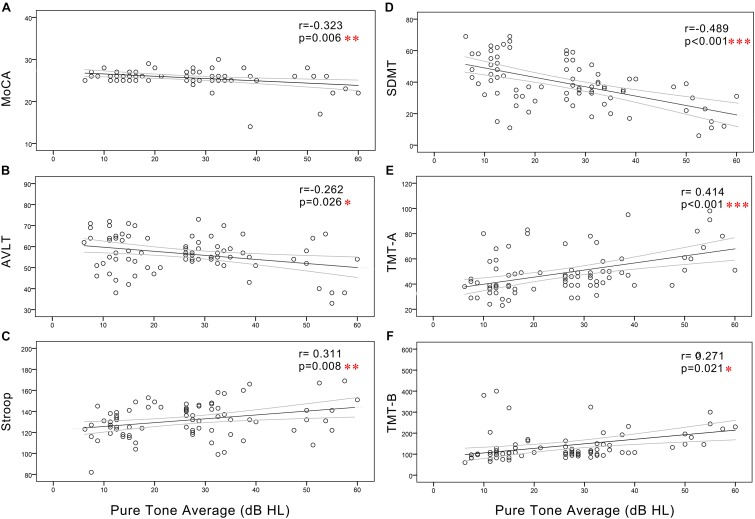
Scatter plots displaying the correlations between PTA and cognitive function in all subjects. Partial correlation analyses (controlling for age, sex, education level, diabetes, smoking, and hypertension) revealed that PTA was positively correlated with **(C)** Stroop (*r* = 0.311, *p* = 0.008), **(E)** TMT-A (*r* = 0.414, *p* < 0.001), and **(F)** TMT-B (*r* = 0.271, *p* = 0.021) and negatively correlated with the **(A)** MoCA (*r* = -0.323, *p* = 0.006), **(B)** AVLT (*r* = -0.262, *p* = 0.026), and **(D)** SDMT (*r* = -0.489, *p* < 0.001). Gray curves: 95% confidence interval of the line of best fit.

**FIGURE 4:**
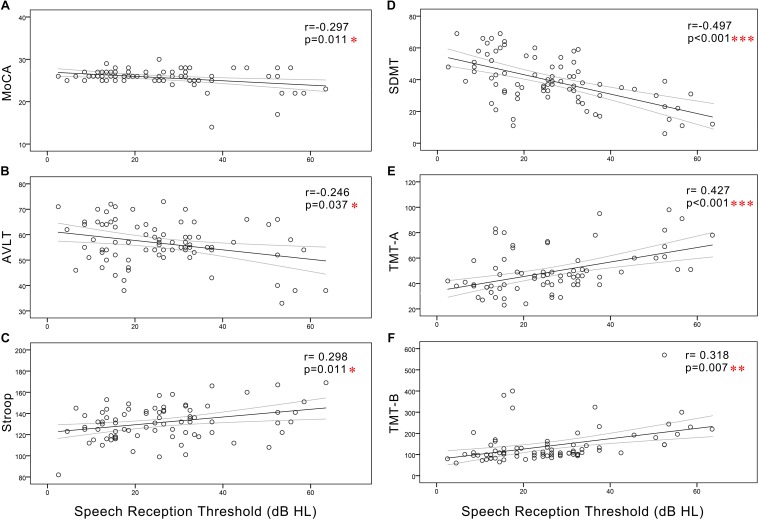
Scatter plots displaying the correlations between SRT and cognitive function in all subjects. Partial correlation analyses (controlling for age, sex, education level, diabetes, smoking, and hypertension) revealed that SRT was positively correlated with **(C)** Stroop (*r* = 0.298, *p* = 0.011), **(E)** TMT-A (*r* = 0.427, *p* < 0.001), and **(F)** TMT-B (*r* = 0.318, *p* = 0.007) and negatively correlated with the **(A)** MoCA (*r* = –0.297, *p* = 0.011), **(B)** AVLT (*r* = –0.246, *p* = 0.037), and **(D)** SDMT (*r* = –0.497, *p* < 0.001). Gray curves: 95% confidence interval of the line of best fit.

In all subjects, Pearson correlation analyses revealed that PTA (Figure 3) was positively correlated with Stroop (*r* = 0.311, *p* = 0.008), TMT-A (*r* = 0.414, *p* < 0.001), and TMT-B (*r* = 0.271, *p* = 0.021) and negatively correlated with the MoCA (*r* = -0.323, *p* = 0.006), AVLT (*r* = -0.262, *p* = 0.026), and SDMT (*r* = -0.489, *p* < 0.001). SRT (Figure 4) was positively correlated with Stroop (*r* = 0.298, *p* = 0.011), TMT-A (*r* = 0.427, *p* < 0.001), and TMT-B (*r* = 0.318, *p* = 0.007), and negatively correlated with the MoCA (*r* = -0.297, *p* = 0.011), AVLT (*r* = -0.246, *p* = 0.037), and SDMT (*r* = -0.497, *p* < 0.001).

**Table 2:** Correlations between audiological (PTA/SRT) and cognitive outcomes (verbal/non-verbal tests).

		Verbal tests			Non-verbal tests		
		MoCA	AVLT	Stroop	SDMT	TMT-A	TMT-B
**All subjects**							
PTA	**r**	**–0.323**	**–0.262**	**0.311**	**–0.489**	**0.414**	**0.271**
	**p**	**0.006**	**0.026**	**0.008**	**<0.001**	**<0.001**	**0.021**
SRT	**r**	**–0.297**	**–0.246**	**0.298**	**–0.497**	**0.427**	**0.318**
	**p**	**0.011**	**0.037**	**0.011**	**<0.001**	**<0.001**	**0.007**
**PC group**							
PTA	**r**	**–0.376**	**–0.536**	0.205	**–0.532**	**0.572**	**0.494**
	**p**	**0.022**	**<0.001**	0.223	**<0.001**	**<0.001**	**0.002**
SRT	**r**	**–0.373**	**–0.574**	0.245	**–0.594**	**0.652**	**0.528**
	**p**	**0.023**	**<0.001**	0.143	**<0.001**	**<0.001**	**<0.001**
**NP group**							
PTA	**r**	0.245	**–**0.379	**0.401**	**–0.430**	0.318	0.091
	**p**	0.219	0.051	**0.038**	**0.025**	0.106	0.652
SRT	**r**	**0.457**	**–**0.276	0.297	**–**0.336	0.175	0.291
	**p**	**0.017**	0.164	0.132	0.087	0.383	0.141


*Partial correlation analyses were used and controlled for age, sex, education level, diabetes, smoking, and hypertension. Bold values indicates a statistically significant difference with a *p*-value less than 0.05. PTA, pure tone average; SRT, speech reception threshold; PC, presbycusis; NP, normal PTA; MoCA, montreal cognitive assessment; AVLT, auditory verbal learning test; SDMT, symbol digit modalities test; TMT, trail-making test.*

**FIGURE 5:**
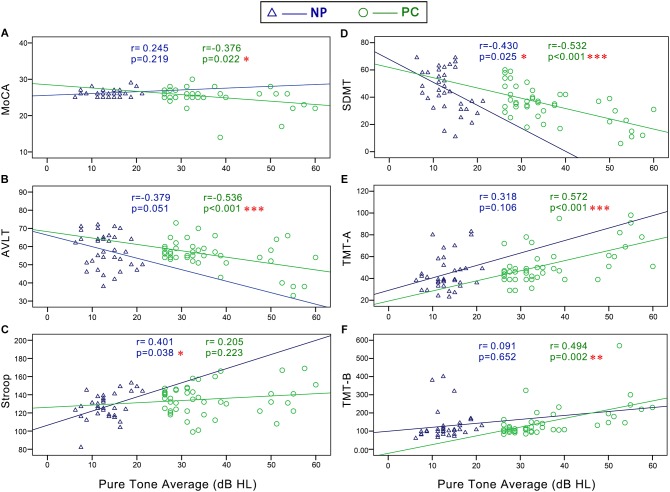
Scatter plots displaying the correlations between PTA and cognitive function in the Normal PTA (NP) group and Presbycusis (PC) group. In the NP group, partial correlation analyses (controlling for age, sex, education level, diabetes, smoking, and hypertension) revealed that PTA was positively correlated with **(C)** Stroop (*r* = 0.401, *p* = 0.038) and negatively correlated with **(D)** SDMT (*r* = –0.430, *p* = 0.025). In the PC group, partial correlation analyses revealed that PTA was positively correlated with **(E)** TMT-A (*r* = 0.572, *p* < 0.001), and **(F)** TMT-B (*r* = 0.494, *p* < 0.001), and negatively correlated with **(A)** MoCA (*r* = –0.376, *p* = 0.022), **(B)** AVLT (*r* = –0.536, *p* < 0.001), and **(D)** SDMT (*r* = –0.532, *p* < 0.001).

**FIGURE 6:**
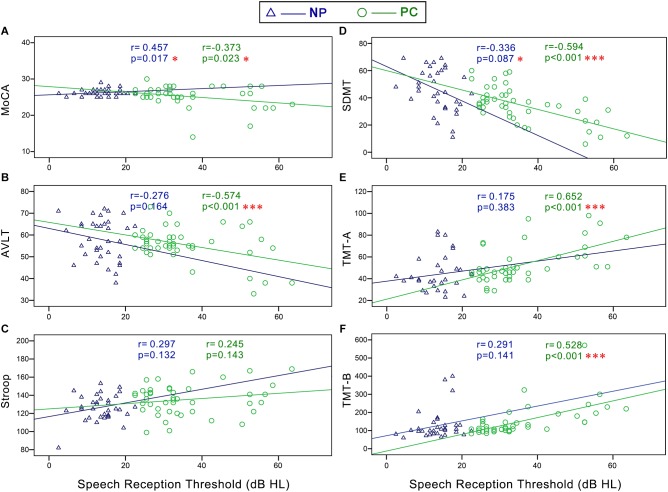
Scatter plots displaying the correlations between SRT and cognitive function in the Normal PTA (NP) group and Presbycusis (PC) group. In the NP group, partial correlation analyses (controlling for age, sex, education level, diabetes, smoking, and hypertension) revealed that SRT was negatively correlated with **(D)** SDMT (*r* = -0.361, *p* = 0.033). In the PC group, Pearson correlation analyses revealed that SRT was positively correlated with **(E)** TMT-A (*r* = 0.585, *p* < 0.001) and **(F)** TMT-B (*r* = 0.602, *p* < 0.001) and negatively correlated with **(A)** MoCA (*r* = -0.300, *p* = 0.046), **(B)** AVLT (*r* = -0.408, *p* = 0.005) and **(D)** SDMT (*r* = -0.622, *p* < 0.001). There were no correlation between SRT and Stroop **(C)** in the NP or PC group.

In the NP group, Pearson correlation analyses revealed that PTA (Figure 5) was positively correlated with Stroop (*r* = 0.401, *p* = 0.038) and negatively correlated with SDMT (*r* = -0.430, *p* = 0.025). SRT (Figure 6) was negatively correlated with SDMT (*r* = -0.361, *p* = 0.033).

In the PC group, Pearson correlation analyses revealed that PTA (Figure 5) was positively correlated with TMT-A (*r* = 0.572, *p* < 0.001), TMT-B (*r* = 0.494, *p* < 0.001), and negatively correlated with MoCA (*r* = -0.376, *p* = 0.022), AVLT (*r* = -0.536, *p* < 0.001), and SDMT (*r* = -0.532, *p* < 0.001). SRT (Figure 6) was positively correlated with TMT-A (*r* = 0.585, *p* < 0.001) and TMT-B (*r* = 0.602, *p* < 0.001) and negatively correlated with MoCA (*r* = -0.300, *p* = 0.046), AVLT (*r* = -0.408, *p* = 0.005), and SDMT (*r* = -0.622, *p* < 0.001).

### Regression Analyses Between Hearing Loss and Cognitive Function (Table 3)

Stepwise Multiple Linear Regression Models were established in all subjects. However, some regression models in NP group and PC group could not be established, so were marked as N/A, presumably because of the small sample size of NP group and PC group.

**Table 3:** Stepwise multiple linear regression models (cognitive measures as dependent variables).

Cognitive	Model I(PTA et al.^∗^ as independent variables)				Model II(SRT et al.^∗^ as independent variables)			
measures	β (95% CI)	R^2^	t	p	β (95% CI)	R^2^	t	p
**All subjects**				
MoCA	–0.050 (–0.079 – –0.021)	0.345	–3.393	0.001	–0.047 (–0.076 – –0.018)	0.336	–3.212	0.002
AVLT	–0.186 (–0.315 – –0.057)	0.143	–2.878	0.005	–0.180 (–0.308 – –0.052)	0.138	–2.792	0.007
Stroop SDMT	0.348 (0.117–0.578)	0.219	2.998	0.004	0.324 (0.091 – 0.557)	0.206	2.771	0.007
	–0.532 (–0.742 – –0.322)	0.322	–5.054	< 0.001	–0.612 (–0.813 – –0.411)	0.312	–6.067	< 0.001
TMT-A	0.561 (0.318 – 0.804)	0.203	4.599	< 0.001	0.571 (0.330 – 0.811)	0.213	4.728	< 0.001
TMT-B	1.611 (0.351 – 2.871)	0.220	2.545	0.013	1.851 (0.589 – 3.113)	0.219	2.920	0.005
**PC group**				
MoCA	–0.096 (–0.160 – –0.033)	0.414	–3.048	0.004	–0.085 (–0.144 – –0.027)	0.434	–2.936	0.005
AVLT	–0.362 (–0.554 – –0.171)	0.343	–3.816	< 0.001	–0.311 (–0.489 – –0.132)	0.347	–3.509	0.001
Stroop SDMT	N/A	N/A	0.919	0.364	N/A	N/A	1.423	0.162
	–0.617 (–0.917 – –0.317)	0.459	–4.154	< 0.001	–0.607 (–0.892 – –0.322)	0.507	–4.303	< 0.001
TMT-A	0.988 (0.588 – 1.388)	0.382	4.999	< 0.001	0.870 (0.529 – 1.210)	0.505	5.160	< 0.001
TMT-B	4.029 (0.260 – 10.341)	0.364	2.122	0.040	4.606 (2.726 – 6.485)	0.362	4.942	< 0.001
**NP group**				
MoCA	N/A	N/A	0.924	0.392	N/A	N/A	1.489	0.146
AVLT	N/A	N/A	1.098	0.280	N/A	N/A	0.874	0.389
Stroop SDMT	1.557 (0.268 – 2.847)	0.129	2.458	0.019	N/A	N/A	N/A	N/A
	–1.687 (–3.090 – –0.283)	0.128	–2.445	0.020	–1.268 (–2.430 – –0.106)	0.104	–2.221	0.033
TMT-A	N/A	N/A	N/A	N/A	N/A	N/A	N/A	N/A
TMT-B	N/A	N/A	N/A	N/A	N/A	N/A	N/A	N/A


*^∗^ Other independent variables include age, sex, education level, diabetes, smoking, and hypertension. PTA, pure tone average; SRT, speech reception threshold; PC, presbycusis; NP, normal PTA; MoCA, montreal cognitive assessment; AVLT, auditory verbal learning test; SDMT, symbol digit modalities test; TMT, trail-making test; levels of anxiety and depression were assessed according to the hospital anxiety and depression scale (HADS); N/A, not available (regression model could not be established).*

In all subjects, PTA was found to be significant predictors of MoCA (β = -0.050, *R*^2^ = 0.345, *p* = 0.001), AVLT (β = -0.186, *R*^2^ = 0.143, *p* = 0.005), Stroop (β = 0.348, *R*^2^ = 0.219, *p* = 0.004), SDMT (β = -0.532, *R*^2^ = 0.322, *p* < 0.001), TMT-A (β = 0.561, *R*^2^ = 0.203, *p* < 0.001), and TMT-B (β = 1.611, *R*^2^ = 0.220, *p* = 0.013). SRT was found to be significant predictors of MoCA (β = -0.047, *R*^2^ = 0.336, *p* = 0.002), AVLT (β = -0.180, *R*^2^ = 0.138, *p* = 0.007), Stroop (β = 0.324, *R*^2^ = 0.206, *p* = 0.007), SDMT (β = -0.612, *R*^2^ = 0.312, *p* < 0.001), TMT-A (β = 0.571, *R*^2^ = 0.213, *p* < 0.001), and TMT-B (β = 1.851, *R*^2^ = 0.219, *p* = 0.005).

In the PC group, PTA was found to be significant predictors of MoCA (β = -0.096, *R*^2^ = 0.414, *p* = 0.004), AVLT (β = -0.362, *R*^2^ = 0.343, *p* < 0.001), SDMT (β = -0.617, *R*^2^ = 0.459, *p* < 0.001), TMT-A (β = 0.988, *R*^2^ = 0.382, *p* < 0.001), and TMT-B (β = 4.029, *R*^2^ = 0.364, *p* = 0.040). SRT was found to be significant predictors of MoCA (β = -0.085, *R*^2^ = 0.434, *p* = 0.005), AVLT (β = -0.311, *R*^2^ = 0.347, *p* = 0.001), SDMT (β = -0.607, *R*^2^ = 0.507, *p* < 0.001), TMT-A (β = 0.870, *R*^2^ = 0.505, *p* < 0.001), and TMT-B (β = 4.606, *R*^2^ = 0.362, *p* < 0.001).

In the NP group, PTA was found to be significant predictors of Stroop (β = 1.557, *R*^2^ = 0.129, *p* = 0.019) and SDMT (β = -1.687, *R*^2^ = 0.128, *p* = 0.020). SRT was found to be significant predictors of SDMT (β = -1.268, *R*^2^ = 0.104, *p* = 0.033).

## Discussion

The results for older adults in a Han Chinese cohort showed that cognitive function was significantly associated with PTA and SRT. In all subjects, the correlations between non-verbal cognitive scores and SRT were stronger than those between non-verbal cognitive scores and PTA, whereas, the correlations between verbal cognitive scores and PTA were stronger than those between verbal cognitive scores and SRT. Moreover, the correlations between PTA or SRT and cognitive function in the PC group were in principle stronger than those in the NP group. Additionally, there was no significant correlation between SRT and cognitive function in the NP group. To the best of our knowledge, this is the first study to elucidate the relationship between SRT and cognitive function in older adults in a Han Chinese cohort.

Previous studies of older American and Australian adults have reported significant relationships between peripheral hearing loss and global cognitive status (Lin et al., 2011a; Gopinath et al., 2012), executive function (Lin, 2011) and psychomotor speed (Lin et al., 2011a). Consistent with these findings, our study indicated, among older adults in a Han Chinese cohort, peripheral hearing, indexed by the four-frequency PTA in the better hearing ear, was significantly related to global cognitive status (MoCA) and multiple domains of cognitive performance, such as psychomotor speed (SDMT), executive function (TMT-A and TMT-B), attention (Stroop) and verbal learning and memory (AVLT). Our results were robust in analyses that accounted for confounders such as age, sex and education level. The strong connection between hearing loss and cognitive decline has been explained by a hypothesis of cognitive resource depletion in older adults (Wayne and Johnsrude, 2015). The hypothesis suggests that hearing loss can lead to increased cognitive resources for understanding acoustically degraded speech. In one functional magnetic resonance imaging study, patients with PC showed greater blood oxygen level-dependent activation responses to acoustic stimuli in the temporal lobes, demonstrating compensatory recruitment of the cortical regions involved in cognitive functions (Ouda et al., 2015). Furthermore, our results demonstrated that the correlations between multiple domains of cognitive performance (e.g., SDMT and TMT) and hearing loss were stronger than those between global cognitive status (e.g., the MoCA) and hearing loss. Tests of multiple domains of cognitive performance are more challenging than the MoCA. Hearing loss could result in a smaller pool of cognitive resources being available for complex cognitive processes, such as psychomotor speed and executive control (Kahneman, 1973).

Pure tone average is considered to be a measure of the auditory periphery because the detection of pure tones relies on cochlear transduction and neuronal afferents to brainstem nuclei, without requiring significant higher auditory cortical processing (Pickles, 1988). It has long been accepted that PC has two components: one that represents aging of the auditory periphery, particularly the inner ear, and another related to aging of the central auditory system. Therefore, in terms of the association between hearing loss and cognitive function, we should take into account the state of both the peripheral hearing and the central auditory system. SRT is used to assess speech detection, and the SRT findings point to a central component of PC. In our study, cognitive function was significantly associated with SRT among older adults. Moreover, in all subjects, the correlations between non-verbal cognitive scores and SRT were stronger than those between non-verbal cognitive scores and PTA, whereas, the correlations between verbal cognitive scores and PTA were stronger than those between verbal cognitive scores and SRT. Therefore, our findings indicated that SRT could be an important auditory test for exploring cognitive decline in PC and could complement PTA. In addition, degraded verbal communication associated with hearing loss may confound cognitive testing. Therefore, non-verbal tests, such as the SDMT and TMT, which do not rely heavily on the presentation of verbal information, were also used in our study; furthermore, mild–moderate hearing loss minimally impairs face-to-face communication in quiet environments, particularly in the setting of testing by experienced examiners (Gordon-Salant, 2005).

Only one study has reported the relationship between SRT and cognitive function in older people (Nkyekyer et al., 2019). In that study, several cognitive domains were associated with SRT, but only the Stroop measure was associated with PTA. In our study, the sample size is larger than that study, and subjects were further divided into presbycusis and normal PTA groups. Moreover, a Han Chinese cohort and a different set of cognition measures were assessed in our study, and we controlled age, sex, education level and other risk factors for cognitive decline in investigating the correlations between cognition and PTA/SRT.

Despite a growing body of literature documenting significant relationships between peripheral hearing and cognition (Gordon-Salant and Fitzgibbons, 1997; Pichora-Fuller and Singh, 2006; Akeroyd, 2008; Lin, 2011; Grassi and Borella, 2013), a few studies have reported negative results (Gates et al., 2002; Idrizbegovic et al., 2011). For example, one study reported no relationship between peripheral hearing and changes in global mental status over 4 years, after adjusting for covariates (Lin et al., 2004). To determine the extent to which the inclusion of many subjects with NP diffused the effects of peripheral hearing loss on measures of cognition, we repeated our correlation analyses within the NP and PC groups. The correlations between PTA or SRT and cognitive function in the PC group were stronger than those in the NP group. Additionally, there was no significant correlation between SRT and cognitive function in the NP group. This suggests that the significant relationship found between hearing and cognition was mainly driven by the older adults with PC. Therefore, some negative results may be attributed to the inclusion of a sample of older adults with NP.

There are several limitations to the present study. First, as this was a cross-sectional study, although correlations between cognitive impairment and hearing loss were found in older adults, the causation is still unknown. Second, the sample size in the study was relatively small. Third, PTA was only measured at four frequencies in the better ear, and it is likely that many subjects would have had high-frequency sensorineural hearing loss that was not captured in our study due to the limited range of frequencies tested.

## Conclusion

Our findings indicate that cognitive function is significantly associated with PTA and SRT in older adults in a Han Chinese cohort. Moreover, the correlations between PTA or SRT and cognitive function in the PC group were in principle stronger than those in the NP group. Based on these findings, we argue that SRT could be an important auditory test for exploring cognitive decline in PC and could complement PTA.

## Ethics Statement

The study was performed in accordance with the Declaration of Helsinki and approved by the institutional review board of the Shandong University. Written informed consent was obtained from all subjects.

## Author Contributions

FG and HW designed the experiments. FR, JL, WM, and LX carried out the experiments. ZF, YA, and BZ analyzed the experimental results. FG and QX assisted. FR and JL wrote the manuscript.

## Conflict of Interest Statement

The authors declare that the research was conducted in the absence of any commercial or financial relationships that could be construed as a potential conflict of interest.
