# Supplementary material for: Hearing Loss and Cognition Among Older Adults in a Han Chinese Cohort
Source: Front Neurosci. 2019 Jun 25;13:632. doi: 10.3389/fnins.2019.00632 (PMC6603159; doi:10.3389/fnins.2019.00632)
Supplement: Supplementary file 1 [file Table_1.DOCX]

Supplementary Material

Hearing Loss and Cognition among Older Adults in a Han Chinese Cohort

**Fuxin Ren^1^†, Jianfen Luo^2,3^†, Wen Ma^4^, Qian Xin^5^, Lei Xu^2,3^, Zhaomin Fan^2,3^, Yu Ai^2,3^, Bin Zhao^1^, Fei Gao^1^*, Haibo Wang^2,3^***

**† These authors contributed equally to this work.**

*** Correspondence: Fei Gao (feigao6262@163.com) , Haibo Wang (whboto11@163.com)**

# Supplementary Figures


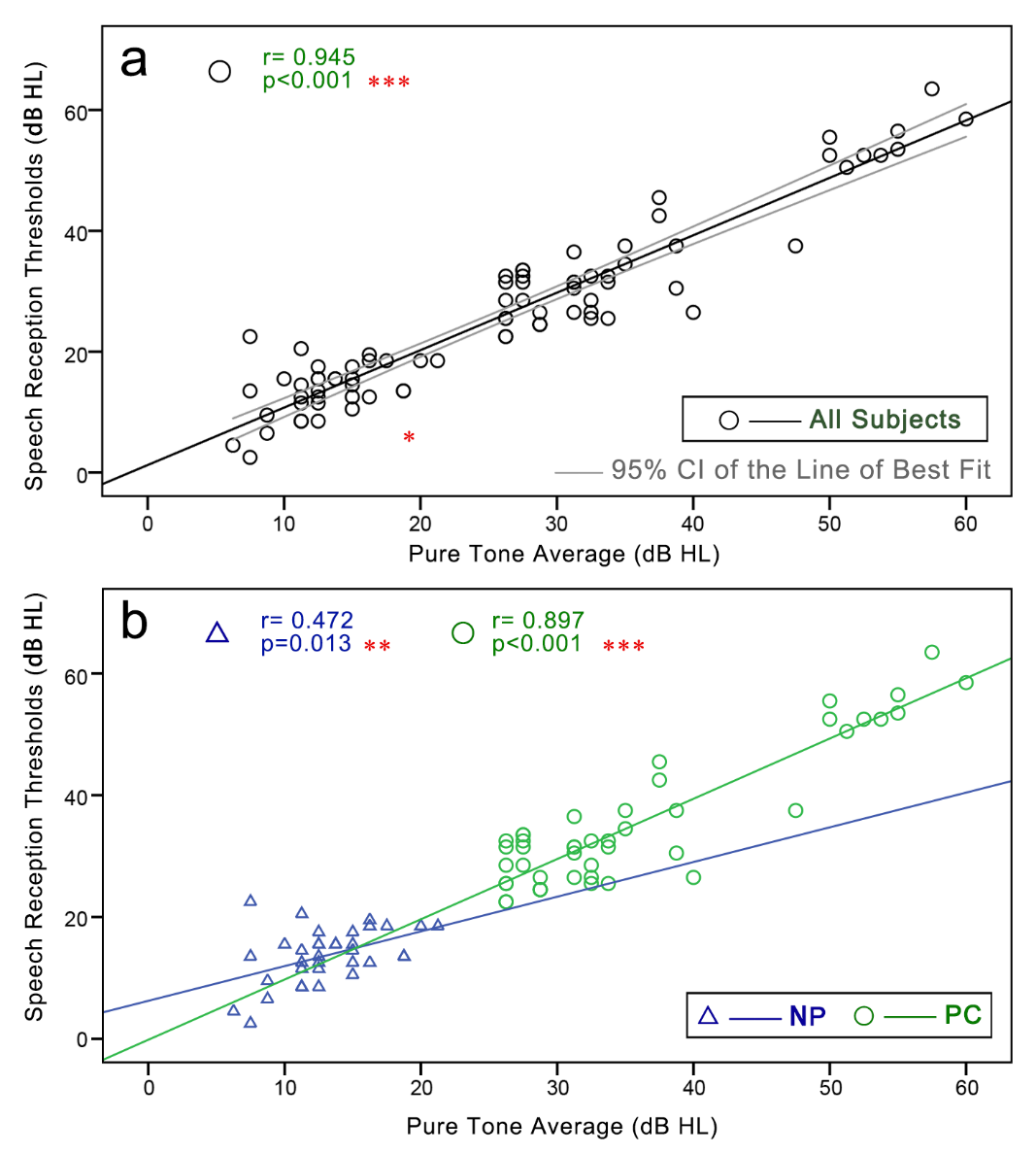


**Figure S1. Scatter plots displaying the correlations between SRT and PTA in all subjects, the Normal PTA (NP) group and Presbycusis (PC) group.**Partial correlation analyses (controlling for age, sex, education level, diabetes, smoking and hypertension) revealed that SRT was positively correlated with PAT in all subjects (r = 0.945, p <0.001) (a), in the NP group (r = 0.472, p =0.013) and in the PC group (r = 0.897, p <0.001) (b).

**Table S1. Correlations between cognitive outcomes.**

|  | | **MoCA** | **AVLT** | **Stroop** | **SDMT** | **TMT-A** | **TMT-B** |
| --- | --- | --- | --- | --- | --- | --- | --- |
| **All subjects** | | | | | | | |
| **MoCA** | **r** | 1.000 | 0.331 | -0.325 | 0.403 | -0.347 | -0.523 |
|  | **p** | -- | 0.004 | 0.005 | <0.001 | 0.003 | <0.001 |
| **AVLT** | **r** | 0.331 | 1.000 | -0.386 | 0.505 | -0.490 | -0.512 |
|  | **p** | 0.004 | -- | 0.001 | <0.001 | <0.001 | <0.001 |
| **Stroop** | **r** | -0.325 | -0.386 | 1.000 | -0.346 | 0.332 | 0.340 |
|  | **p** | 0.005 | 0.001 | -- | 0.003 | 0.004 | 0.003 |
| **SDMT** | **r** | 0.403 | 0.505 | -0.346 | 1.000 | -0.647 | -0.604 |
|  | **p** | <0.001 | <0.001 | 0.003 | -- | <0.001 | <0.001 |
| **TMT-A** | **r** | -0.347 | -0.490 | 0.332 | -0.647 | 1.000 | 0.672 |
|  | **p** | 0.003 | <0.001 | 0.004 | <0.001 | -- | <0.001 |
| **TMT-B** | **r** | -0.523 | -0.512 | 0.340 | -0.604 | 0.672 | 1.000 |
|  | **p** | <0.001 | <0.001 | 0.003 | <0.001 | <0.001 | -- |
| **PC Group** | | | | | | | |
| **MoCA** | **r** | 1.000 | 0.468 | -0.384 | 0.648 | -0.508 | -0.739 |
|  | **p** | -- | 0.004 | 0.019 | <0.001 | 0.001 | <0.001 |
| **AVLT** | **r** | 0.468 | 1.000 | -0.196 | 0.396 | -0.603 | -0.558 |
|  | **p** | 0.004 | -- | 0.244 | 0.015 | <0.001 | <0.001 |
| **Stroop** | **r** | -0.384 | -0.196 | 1.000 | -0.226 | 0.242 | 0.386 |
|  | **p** | 0.019 | 0.244 | -- | 0.179 | 0.149 | 0.018 |
| **SDMT** | **r** | 0.648 | 0.396 | -0.226 | 1.000 | -0.671 | -0.600 |
|  | **p** | <0.001 | 0.015 | 0.179 | -- | <0.001 | <0.001 |
| **TMT-A** | **r** | -0.508 | -0.603 | 0.242 | -0.671 | 1.000 | 0.658 |
|  | **p** | 0.001 | <0.001 | 0.149 | <0.001 | -- | <0.001 |
| **TMT-B** | **r** | -0.739 | -0.558 | 0.386 | -0.600 | 0.658 | 1.000 |
|  | **p** | <0.001 | <0.001 | 0.018 | <0.001 | <0.001 | -- |
| **NP Group** | | | | | | | |
| **MoCA** | **r** | 1.000 | -0.079 | 0.155 | -0.140 | 0.220 | 0.069 |
|  | **p** | -- | 0.696 | 0.440 | 0.487 | 0.270 | 0.732 |
| **AVLT** | **r** | -0.079 | 1.000 | -0.476 | 0.694 | -0.335 | -0.539 |
|  | **p** | 0.696 | -- | 0.012 | <0.001 | 0.087 | 0.004 |
| **Stroop** | **r** | 0.155 | -0.476 | 1.000 | -0.335 | 0.373 | 0.173 |
|  | **p** | 0.440 | 0.012 | -- | 0.088 | 0.056 | 0.388 |
| **SDMT** | **r** | -0.140 | 0.694 | -0.335 | 1.000 | -0.606 | -0.658 |
|  | **p** | 0.487 | <0.001 | 0.088 | -- | 0.001 | <0.001 |
| **TMT-A** | **r** | 0.220 | -0.335 | 0.373 | -0.606 | 1.000 | 0.723 |
|  | **p** | 0.270 | 0.087 | 0.056 | 0.001 | -- | <0.001 |
| **TMT-B** | **r** | 0.069 | -0.539 | 0.173 | -0.658 | 0.723 | 1.000 |
|  | **p** | 0.732 | 0.004 | 0.388 | <0.001 | <0.001 | -- |

**Notes:** Partial correlation analyses were used and controlled for age, sex, education level, diabetes, smoking, hypertension.

**Abbreviations:** PTA, pure tone average; PC, presbycusis; NP, normal PTA; MoCA, Montreal Cognitive Assessment; AVLT, Auditory Verbal Learning Test; SDMT, Symbol Digit Modalities Test; TMT, Trail-Making Test.
